# Trust and task-sharing: the acceptability of nurses and peer educators in PrEP delivery among sexually and gender-diverse adolescents in Brazil

**DOI:** 10.21203/rs.3.rs-9040931/v1

**Published:** 2026-04-15

**Authors:** Cristiane Spadacio, Lorruan Alves dos Santos, Sarah MacCarthy, Katia Bruxvoort, Inês Dourado, Laio Magno, Paula Massa, Eudardo Araújo de Oliveira, Eliane Aparecida Sala, Marcia Thereza Cavalcanti Couto, Alexandre Grangeiro

**Affiliations:** Universidade de São Paulo; Universidade de São Paulo; University of Alabama; University of Alabama; Universidade Federal da Bahia; Universidade do Estado da Bahia; Universidade de São Paulo; Universidade de São Paulo; Universidade de São Paulo; Universidade de São Paulo; Universidade de São Paulo

**Keywords:** Pre-exposure prophylaxis (PrEP), Adolescents, HIV prevention, Peer educators, Nursing, Long-acting injectable PrEP, Key populations, Brazil, Qualitative research

## Abstract

**Objectives:**

To examine the acceptability of oral and long-acting injectable PrEP (LAI-PREP) delivery by nurses and peer educators among sexual and gender diverse adolescents in Brazil.

**Design:**

A qualitative study using semi-structured interviews and reflexive thematic analysis.

**Setting:**

Community-based PrEP services in São Paulo and Salvador, Brazil, conducted within the PrEP15–19 demonstration study.

**Participants:**

Twenty PrEP clients aged 15–23 years (16 Black, 4 White; 14 cisgender men, 5 transgender or gender-diverse individuals) and nine healthcare staff (5 nurses, 2 peer educators, 2 physicians).

**Results:**

Trust emerged as the foundational element of PrEP engagement. Clients perceived nurses and physicians as having equivalent competence for prescribing PrEP though nurses were described as providing more comprehensive information. Support for peer-led prescription was mixed; most participants preferred peer involvement to remain focused on education and care navigation rather than clinical procedures. While LAI-PrEP was viewed positively, its technical requirements reinforced a preference for nurse-led delivery over peer involvement.

**Conclusions:**

Nurses are widely accepted as PrEP prescribers, while peer educators are valued primarily for their complementary, supportive roles. The introduction of LAI-PrEP may reinforce medicalized care models. To successfully implement differentiated PrEP delivery, programs must address professional hierarchies and prioritize the development of interdisciplinary teams.

## BACKGROUND

In Brazil, the HIV epidemic is concentrated in key populations, such as men who have sex with men (MSM), and transgender women (TGW) [1,2]. Evidence points to a sharper increase in infections in adolescents and young people [3], especially in such groups[4]. Surveillance data from 2024 show that among Brazilian MSM aged 15–24 years, the estimated HIV incidence per 100,000 individuals increased by 75% [5].

Pre-exposure prophylaxis (PrEP) represents a critical innovation in HIV prevention and Brazil has been a global leader in HIV response, first through universal antiretroviral therapy (ART) access and recently through PrEP implementation[4]. Oral PrEP is a highly effective HIV prevention strategy [6], particularly for sexual and gender diverse (SGD) adolescents in Brazil. Its impact is bolstered by the National Health System (SUS), which provides the medication at no cost to individuals aged 15 and older. [7]. Furthermore, in Brazil, according to the Child and Adolescent Statute (ECA) [8], adolescents are entitled to privacy and confidentiality in the healthcare system. Parental authorization is not required for consultations. The ECA ensures their right to comprehensive health protection and access to care.

Unlike oral PrEP, which requires daily adherence or an on-demand regimen, long-acting injectable PrEP (LAI-PrEP) represents a clinical innovation administered via intramuscular or subcutaneous injections at extended intervals, such as every two to six months. These modalities present distinct implementation challenges; for instance, LAI-PrEP requires more sensitive diagnostic protocols to detect recent HIV infections and trained health professionals to ensure correct administration. Consequently, the WHO emphasizes the need for research into service delivery strategies that enhance acceptance, access, and sustained engagement among SGM adolescents [9].

Service delivery models and provider roles are pivotal to the success of HIV prevention. While PrEP provision in Brazil was historically PrEP restricted to physicians, the 2020 national guidelines expanded this scope to include nurses, dentists and pharmacists. Subsequently, evidence suggests that nurse-led models facilitate greater engagement in PrEP care continuum, leveraging their clinical expertise in antiretroviral management to foster high levels of user acceptance [10]. Consequently, both the United Nations and the Brazilian Ministry of Health advocate for the full integration of nurses into PrEP delivery to decentralize care and improve service accessibility [7].

There is growing interest in leveraging “peers”, including peer educators (information dissemination), peer navigators (linkage to care) or lay health workers (service delivery) – to simplify PrEP provision and enhance retention in care[11]. Emerging evidence highlights the efficacy of PrEP prescription and clinical follow-up conducted by trained lay professionals or peers [12,13]. These findings support the integration of peer-led models to improve outreach among key populations, such as SGD adolescents, thereby fostering broader PrEP accessibility and sustained adherence [12].

Further research is essential to evaluate the acceptability of PrEP provision by nurses and peer educators and to understand how adolescents accustomed to traditional, physician-centred models perceive these shifts in care. Identifying how diverse providers can dismantle barriers to access and retention is critical, particularly when compared to conventional clinical settings. This study analyzes the perceptions of SGD adolescents who received daily oral PrEP care through nurses and peer educators in community health services. Furthermore, it examines user perceptions regarding evolving guidelines, specifically the integration of nurses into oral PrEP delivery and the potential for nurse- and lay-led delivery of LAI-PrEP.

## METHODS

This qualitative study engaged healthcare staff such as physicians, nurses, peer educators, and PrEP clients from the PrEP15–19 demonstration project, which evaluated the effectiveness of daily oral PrEP among adolescents MSM and *travestis’* and *transsexualwomena* ≥ *d*15 – 19[14, 15]. *InBrazil, theterms*travestis’ and `transsexual’ are commonly used by the communities themselves, expressing distinct political perspectives. Therefore, to acknowledge the Brazilian context, we use the term travestis and transsexual women (TrTW) in this article to reference our study participants and use TGW to refer to transgender women more broadly in the international literature.

We analyzed interviews conducted in two contrasting urban settings: Salvador, Bahia, one of Brazil’s poorest state capitals, and São Paulo, (São Paulo), the country’s wealthiest city.

Interview participants were selected via purposive sampling to ensure representation across key categories. Among the clients (n=20), we included both active oral PrEP users and those, ensuring diversity in self-reported gender identity and sexual orientation. Healthcare staff interviews included five prescribing nurses, two peer educators (tasked with recruitment, navigation and user support), and two physicians.

Specific interview guides were developed and applied for each participant group (healthcare staff and clients). The interview guides are provided in the supplementary material (Additional file 1 and Additional file 2). Healthcare staff were asked the following domains: i) socioeconomic and training-related identification, including professional trajectory and motivations for working in HIV/STI prevention; ii) perceptions of the HIV epidemic in Brazil, the role of combination prevention, and the values attributed to PrEP, including potential moral judgments toward clients. The interview script also addressed work processes and professional relationships, examining care routines, teamwork practices, interactions with managers, and both positive and challenging experiences in service provision; iii) opinions regarding the expansion of PrEP care and prescribing by nursing professionals and peer educators, considering potential impacts on work organization, interprofessional tensions, required resources, and implications for clients, continuity of care, and user-friendly models of PrEP dispensing; iv) perspectives on LAI-PrEP, including potential benefits and challenges, as well as likely reactions from other professionals and clients.

PrEP clients were asked the following domains: i) socioeconomic identification, covering demographic characteristics, educational background, occupation, household arrangements, living conditions, and aspects related to income and economic dependence; ii) clients’ trajectories of learning about and using PrEP, including how they first heard about it, duration of use, any treatment interruptions, and their overall experiences with PrEP as an HIV prevention strategy; iii) access to health services, the pathways taken to reach PrEP care, perceived barriers and facilitators, and experiences with clinical follow-up, emphasizing interactions with healthcare staff, relationships with other clients, any experiences of stigma or discrimination, and perceptions about receiving care in non-specialized services; iv) the guide also examined the acceptability of PrEP care and prescribing by nursing professionals, exploring trust, comparisons with medical care, continuity of care, and potential advantages associated with nurse-led PrEP management; v) investigated experiences and perceptions related to follow-up by peer educators, including rapport, support, and opinions on the possibility of these professionals performing tasks currently assigned to nurses. Finally, the interviews explored views on LAI-PrEP and the acceptability of receiving it from nurses or peer educators, including perceived benefits and challenges identified by clients.

After explicit written consent was obtained, all interviews were conducted by experienced researchers previously trained and familiar with the interview script. We conducted the interviews virtually through an online video conferencing platform. Financial compensation was offered for expenses related to participation. The interview audios were transcribed and reviewed, and the content was inductively categorized via MAXQDA software version 2022. Fictitious names were used to ensure anonymity. In the excerpts presented in the results section, we used the codename from the table, followed by an indication of whether the participant is a client or a professional.

A thematic analysis was carried out by two researchers, using the assumptions of Baun & Clarke [16] on the basis of pre-analysis procedures of the collected material, data mining, inference, and interpretation of the results. The process was carried out in five stages: 1) a first reading to provide a holistic overview and capture particularities of the interviews; 2) definition of the emerging empirical categories and content categorization; 3) preliminary synthesis; 4) literature review and discussion of the findings obtained in the preliminary synthesis; and 5) final interpretive synthesis.

The research was approved by the Ethics and Research Committee of the Faculty of Medicine of the University of São Paulo and the Collective Health Institute of the Federal University of Bahia, Brazil (CAAE: 89993018.9.0000.0065 and opinion number: 5,603,541). All participants provided written informed consent for participation and publication of anonymized quotations.

This study followed the guidelines of the COREQ (Consolidated Criteria for Reporting Qualitative Research) protocol, which consists of 32 items designed to ensure transparency and completeness in the reporting of qualitative studies (Supplementary File 2) [17].

## RESULTS

Client interviews were equally distributed between Salvador and São Paulo. At the time of the interview, 14 participants were aged 15–19, and 6 were aged 20–23. Regarding race, 16 identified as black (including both brown and black categories), and 4 as white. The sample included 14 cisgender men and 5 TrTW. In terms of sexual orientation, 11 participants identified as gay, 5 as heterosexual, and 2 as bisexual; additionally, one participant identified as “questioning”, and one as “transgender” when describing their orientation.

We interviewed 5 healthcare staff in São Paulo and 4 in Salvador, aged 25 to-45. The racial composition was evenly split between black (4) and white (4). Regarding gender identity and sexual orientation, 4 cisgender women, 3 cisgendermen, and 1 transgender person; 3 identified as heterosexual, 2 as bisexual, and 1 participant who declined to state. In São Paulo, 3 nurses were interviewed (one of whom also served as a service manager), 1 physician, and 1 peer educator. The Salvador team consisted of 2 nursing professionals, 1 physician, and 1 peer educator responsible for navigation.

Detailed information is available in Table 1.

Empirical categories emerged from interviews: the overarching role of trust; updated guidelines - the role of nurses; evolving guidelines - the role of peers; general perceptions of LAI-PrEP; modes of delivery for LAI-PrEP: nurses and peers.

Regarding the category of the overarching role of trust, interviewees in this study highlighted the strong confidence they placed in both nurses and peer educators. This trust was grounded in two main areas: technical training - clients perceived adequate technical competence, such as the ability to prescribe PrEP, order and interpret clinical exams, and conduct physical evaluations. And, interpersonal qualities.

Nurses were frequently described as approachable, friendly, and welcoming, which made users feel more comfortable initiating and adhering to PrEP. Peer educators were valued for their closeness to the communities most affected by HIV, which helped to foster trust and a sense of shared experience.

Clients also point to trust in nurses and peer educators due to a perception of previous adequate health training to perform procedures related to oral PrEP, such as prescribing, requesting and analyzing clinical exams, and performing physical evaluations. Moreover, users highlighted nurses’ friendliness and welcoming nature in adhering to PrEP; and peers for their proximity to the target population.

But I think it’s totally fine for a nurse to prescribe PrEP. I think it conveys the same, I don’t know, I’d even say more, because there the nurses themselves are the one who does the blood sample collection, in the project. So, it already creates that bond: they go and do the blood sample collection, take your blood, it just gives off that feeling of trust (João, professional).

I found it quite, like friendly. It was quite welcoming too [nursing care] (Henrique, client).

Clients’ perceptions regarding **the role of nurses in PrEP delivery** were overwhelmingly positive, especially concerning the expanded scope of prescribing authority. Considering the perspective of clients, they perceived no difference in the competence, safety, and quality of care between nurses and physicians when it came to prescribing oral PrEP.

There was no difference [being treated by doctors or nurses] since I had the same level of trust. In this regard, nothing would change for me. Whether a nurse or a doctor, it would not change anything (Irene, client).

I believe that if I adhere to some test, I do not know, but I believe that a doctor can do more things. However, in PrEP clinical care, I do not see any difference [between nurses and doctors] (Amon, client).

Clients also compared the roles of nurses and physicians, underscoring the different communication styles of each group. Nurses were described as more attentive and thorough, often taking more time to explain procedures and exams. Physicians, by contrast, were seen as more concise and “direct,” though this style was also valued because it often came with clear referrals and instructions about potential side effects. Importantly, participants saw no difference in the perceived competence of nurses versus physicians when it came to prescribing PrEP.

I believe that if I adhere to some test, I do not know, but I believe that a doctor can do more things. However, in PrEP clinical care, I do not see any difference [between nurses and doctors] (Amon, client).

As I told you, the nurse ended up providing much more information, you know? She explained much more of the procedure in a nutshell. The doctor said, look, everything’s fine, you can rest assured. In addition, bye. In addition, bye, that is it. That was basically it (Ramon, client).

From the perspective of healthcare professionals, both physicians and nurses acknowledged that expanding PrEP prescribing authority to nurses has contributed to progress in HIV prevention, primarily through improved access. Nurses were described as offering user-centred care and demonstrating professional competence, while the increased number of prescribing professionals has enhanced efficiency and responsiveness in PrEP delivery.

I reckon it is an enormous step forward. I believe that we, as nursing professionals, do have professional competence in our practice, even on the basis of what we learn in the undergraduate curriculum. I consider it a very good step. I think it facilitates access owing to the holistic view that nurses tend to have, which ends up easing the understanding to move away from that diagnostic-centered environment and understand that there is a vulnerability; instead, I comprehend the individual as a unique case, understanding the increased vulnerabilities, their routine for taking this medication, and their understanding of why they are taking this medication (Ricco, professional).

Several interviewees noted that this model was especially beneficial in remote areas, where physicians are less available, citing examples from Salvador in particular as evidence of expanded reach.

In the countryside, many times, there is not even a doctor. Therefore, it is extremely necessary to have nurses who understand, can write this prescription, and raise awareness among users of the importance of using PrEP. Most importantly, nurses can show that it works and that people can actually obtain PrEP. (Nice, professional).

Nevertheless, all the clients were well attended to by both professionals and faced no issues with PrEP prescriptions. Therefore, similar perceptions of care safety and quality prevailed, with the assumption of well-trained professionals.

There was no difference [being treated by doctors or nurses] since I had the same level of trust. In this regard, nothing would change for me. Whether a nurse or a doctor, it would not change anything (Irene, client).

Responses regarding peer-led oral PrEP delivery were varied. Participants specifically questioned the clinical competency of peers in conducting medical procedures, including rapid diagnostic testing and specimen collection.

Well, I would say it is fine to a certain extent, like, to talk, explain the risks, those things. However, then, I do not think it makes much sense for a peer educator, for example, to collect my blood to do a blood test. You know? (Guto, client)

Others indicated they would feel comfortable with peer prescribing if peers received proper biomedical training and worked in collaboration with medical staff who could manage clinical complications. For clients, peer prescribing was viewed as a way to reduce long wait times for services, which they saw as a practical advantage.

Wonderful! [about the possibility of being seen by a peer educator] Because sometimes we are restricted to the nurse, right? Sometimes nurses are unavailable, and we have to wait for their arrival so they can prescribe the medication. In that sense, I believe that having peer educators would be wonderful (Guiga, client).

Health professionals also expressed ambivalence. On the one hand, they recognized the unique potential of peer educators to connect with users and promote access by bringing services closer to lived realities.

I consider that many times the educator can have more contact with the participants, with the young people, with the patients, right, anyway, where there truly may be more openness to certain, right, approaches that often the health professional may not have, for reasons of, well, right, you know that there are barriers there, right, placed (Samara, professional).

On the other hand, concerns about technical competence and ethical standards were raised, with training identified as a non-negotiable condition for peers to take on expanded responsibilities.

I believe they indeed need efficient training to understand the entire process to ensure their confidence when prescribing (Eleonora, professional).

Some providers went further, suggesting that resistance to peer involvement was rooted in broader professional hierarchies. According to participants, physicians’ reluctance to share prescribing authority reflected entrenched notions of exclusive medical expertise, reinforced by dynamics of class and race that shape who holds power within the health system.

I think it would be a massive taboo to break […] I think it would be challenging because when we consider health professionals, the vast majority do not engage with reality. […] They are rich, white people who live in a bubble and do not have much interest in issues other than their own. In that sense, if someone suddenly stresses, “Look, there’s going to be someone from the community, he finished high school, and he’s going to prescribe PrEP with us, he’s going to be part of our team.” That will generate a fuss, including from the councils and agencies themselves, because unfortunately, only the worst people represent us - and I say this as a doctor (Diego, professional).

When we moved on from the experience with oral PrEP and pointed to reflections on **general perceptions of LAI-PrEP**, most clients reported little or no prior knowledge of injectable PrEP. After receiving information, however, they tended to evaluate the option positively. Benefits frequently mentioned included reduced pill burden, fewer worries about adherence and, particularly for TrTW already managing hormone therapy, the convenience of reducing the number of daily medications. LAI-PrEP was also seen as requiring fewer clinic visits, minimizing disruption to work or school schedules, and reducing stigma or discrimination by eliminating the need to carry pills.

At the same time, clients voiced several concerns. Chief among these were the requirement to continue oral PrEP for a year after stopping injections, the perception that LAI-PrEP might cause more serious side effects or injection-site injuries, and fears of pain from the injection itself.

The category on **modes of delivery for LAI-PrEP by nurses and peers** reveals that respondents generally expressed high levels of comfort with nurses prescribing and administering the injections. This trust was rooted in their prior experiences with nurses in the context of oral PrEP, where nurses were seen as reliable, approachable, and technically competent. For many participants, the involvement of nurses offers reassurance and a sense of continuity as new PrEP modalities emerge.

Interview: In that case, what do you think about injectable PrEP, i.e., receiving injectable PrEP from nurses? Participant: Easy as a pie, yes. As I told you, I trust them a lot, you know? And if they administer it, it would be great.

In a way, nurses already have greater ease, e.g., they already have a recommendation for this, that is, injecting into a vein, if I’m not mistaken. Therefore, if you miss a vein, you may end up hitting a muscle like that, and then, in a way, I feel more comfortable with nurses. They already have the necessary specialization for this.

In contrast, most clients were less supportive of peer educators prescribing or administering injectable PrEP. They expressed a preference for these responsibilities to remain with formally trained health professionals, citing the higher stakes and invasiveness of injections compared to oral medication. However, participants strongly valued the role of peer educators in counselling, education, and ongoing support.

Since there is a need for sterilization, equipment handling and use, and other technical capabilities, I can say that I trust nurses more.

Participant: I reckon there is a lack of specialization in this regard, you know? Of piercing an arm, piercing some other part of the body, handling nursing instruments, you know? Although I do not have that much knowledge on the matter, that might be lacking. However, I know that good nurses, because they already worked with this, having taken a course specifically for this, are much more trustworthy. […] Therefore, in this regard, I reckon that the monitoring part and the prescription are easy steps, but the more critical, more technical handling should be done by a nursing professional.

Some indicated that they would accept LAI-PrEP from peers if those peers had received specialized training and were supervised by doctors. This suggests that while peer involvement in PrEP delivery is welcomed, their roles are currently viewed as complementary rather than interchangeable with those of nurses and physicians.

Oh, I do not see any issues with that. At least for me, I do not see any problems if they have training and feel safe doing that.

Bearing in mind that this would follow what we had discussed, with them having instructions, with a professional supervising them in a certain way, I would also feel very comfortable.

Through health professionals’ and peer educators’ lenses, positive and negative aspects can be used to divide perceptions into LAI-PrEP. Among the positive factors, health professionals highlighted LAI-PrEP as an additional possibility to the already available options for HIV prevention. The access promotion to such a technique consists of different perspectives. LAI-PrEP is an alternative to oral medication and its known barriers, such as difficulty swallowing the pill, continuous side effects (in some specific cases), and forgetting to take the medication.

On the other hand, LAI-PrEP with cabotegravir requires bimonthly consultations, whereas oral PrEP tends to last four months, highlighting the easy access to oral PrEP due to more spaced consultations. Thus, it is possible that the perception of a “reduction” in the number of consultations comes from the fact that, with the injection, it will no longer be necessary to use the medication every day.

LAI-PrEP prescription and handling by nursing professionals or peer educators raised safety considerations from the professionals’ perspective, highlighting the need for training nursing professionals and peer educators.

That is still a challenge for me. I believe that prescription safety and pill prescription demand training, and a more in-depth study of how the medication acts in the body and the main adverse side effects of the prescription is needed. Owing to the need for detailed clinical attention, this safety requires specific clinical training on the medication […] Regarding the application, I think more specific training would pave the transition and clear my doubts. Nevertheless, I need more clinical training regarding the prescription of PrEP. Bearing in mind the professionals I have contact with, I am aware that they are new to injectable PrEP. Therefore, further discussions are needed, as are other international studies already pointing to this adherence to injectables. Hence, much more in-depth training is needed (Ricco, professional).

With respect to the aspects reported as negative, some concerns emerged from the professionals’ perspective. The “tail phase” of LAI-PrEP and the barrier to use for cross-dressing and transgender people stood out owing to the frequent use of prostheses and silicone. Less emphatically, the issue of pain related to the injection was also an aspect highlighted by the interviewees.

The statement below from a peer educator about the possible workload for offering injectable PrEP in addition to challenges arising from the new treatment indicates the importance of support in addition to the clinical approach, which is also related to administrative and logistic issues.

Interviewer: Do you think it could create an overload of work in what you already do?

Prof – Peer educator: Surely. Yes, surely. As I told you, we do recruitment work, which takes up most of our hours, and then the on-call work eats up most of our hours. On top of that, we still have to move to retention, which is one of the hardest jobs. In addition, we work with spreadsheets, with SISPREP itself [a data registration platform created by the study], which is quite large; there are many tasks to fill in the information dynamically, i.e., opening WhatsApp, the web, and SISPREP simultaneously.

## DISCUSSION

Our results shed light on both convergences and divergences in how SGD adolescents and healthcare staff perceive the roles of nurses and peer educators in supporting participation and protagonism in the monitoring and delivery of oral and LAI-PrEP.

### The Overarching Role of Trust

The results showed that trust plays a central role among study participants, acting as a mediating element between initial access, the decision to initiate the method, and continuity of care. The narratives indicate that this trust is constructed in a complementary manner through perceived technical competence and the relational qualities of nurses and peer educators. The perception that nursing professionals have adequate training to prescribe PrEP, request and interpret clinical examinations, and perform physical assessments reinforces the legitimacy of care and reduces uncertainties regarding the safety of the method. International evidence demonstrates that nurse-led PrEP models are clinically viable, safe, and effective—particularly when supported by clear protocols and appropriate supervision—and that they also expand access in contexts of physician shortages or high demand within healthcare services [18].

Beyond the technical dimension, trust emerges as strongly associated with interpersonal interactions. Clients described nursing care as welcoming, approachable, and non-judgmental—qualities that foster relationship building and create a sense of comfort when discussing sensitive issues related to sexuality, HIV risk, and PrEP side effects. The role of peer educators complements this process by operating as a vector of community-based trust, grounded in territorial proximity and shared experience with populations most affected by HIV. The literature on peer-led interventions highlights that this closeness helps reduce barriers related to stigma, fear of discrimination, and longstanding mistrust of healthcare services, particularly among socially marginalized populations[19].

### Updated Guidelines – The Role of Nurses

Nurses are widely viewed by users and peers as qualified professionals possessing the technical competencies required for PrEP assessment, prescription, and clinical follow-up. Specifically, nurses are perceived by most clients as capable and possessing the technical skills necessary to conduct the assessment, prescription, and clinical monitoring of oral or LAI-PrEP. Furthermore, our results reveal that health professionals consider the inclusion of nurses as prescribers and coparticipants in PrEP care as essential to increasing the capacity of PrEP services, thus making prophylaxis available to more users, a perception corroborated by adolescents.

The discussion about the participation of nursing professionals in prescribing PrEP has been on the agenda for expanding and democratizing access to PrEP, especially owing to the WHO’s recognition that these professionals have the requirements to carry out this monitoring. This recognition is supported, above all, by the successful experience of these professionals in prescribing antiretroviral therapy, which has considerably increased the number of people with HIV in treatment, representing, in some situations, the ideal care model for users [10,20]. In Brazil, nurses have been able to prescribe postexposure prophylaxis for HIV (PEP) and PrEP within the scope of the SUS since July 2020.

The vast majority of adolescents positively evaluated the PrEP monitoring provided by nurses, often describing the care provided by these professionals as more informative and detailed than that provided by physicians. Similar results were reported in a study investigating the feelings and experiences of PrEP users led by nurses in Canada[20]. In this study, the participants reported that the personalized approach of nurses provided a sense of security about the care and support they received. Furthermore, according to the authors, participants often wished that nurses would not only help them with PrEP and other aspects of their lives but also take responsibility for fixing the entire health system [20].

### Evolving Guidelines – The Role of Peers

Peer educators tend to be recognized and accepted primarily as supportive contributors within the PrEP care process. A systematic review of evidence from studies on PrEP delivery among US adolescents and young adults demonstrated that personalized navigation and case management are consistently effective strategies, particularly for marginalized youth facing multiple structural barriers [21].

Even when they operate under the supervision of clinical professionals, concerns persist regarding the scope and safety of the care they provide. Over time, peer educators may obtain greater autonomy once fully integrated and supported within multidisciplinary teams, operating under the logic of medicalization anchored in biomedical knowledge. However, apprehension persists regarding delegating care responsibilities to peers, reinforcing ongoing limits to their participation within PrEP delivery models.

### Evolving Guidelines – General Perceptions of LAI-PrEP

Perceptions of health professionals, peer educators, and clients were generally positive regarding the advantages of LAI-PrEP: no need to remember daily pills, fewer visits to health services, and reduced exposure to stigmatizing environments. Notably, clients often perceive LAI-PrEP as requiring fewer clinic visits, despite protocols mandating bimonthly appointments for clinical reassessment and cabotegravir administration, a higher frequency than the quarterly follow-ups required for oral PrEP [7]. This perspective suggests that the sense of reduced medical surveillance stems not from the number of appointments, but from the elimination of daily pill-taking. For many, the transition away from daily oral PrEP alleviates the psychological burden of adherence and the constant reminder of HIV risk.

Concerns regarding side effects and clinical management were prominent. Providers highlighted specific challenges, including ethical and legal considerations for adolescents, contraindications for transgender women with industrial silicone implants, and the necessity of oral PrEP during the pharmacokinetic tail phase. Similarly, clients expressed apprehension regarding adverse effects. These findings align with previous research in the United States and Brazil among sexual and gender-diverse youth, which identified concerns regarding efficacy, access costs, potential side effects, and needle-related discomfort [22–25].

### Evolving Guidelines – Modes of Delivery for LAI-PrEP: Nurses and Peers

Both staff and clients expressed concerns regarding the pharmacological tail of LAI-PrEP and the subsequent requirement for oral PrEP use for at least one year following injectable treatment. This protocol is necessary to prevent the selection of drug-resistant HIV strains, a challenge previously documented in the literature [26,27]that may act as a barrier for high-priority populations. To address this, Meyers et al. emphasize the importance of user counselling, training of healthcare professionals in effective communication, and robust adherence support [27]. Ultimately, prevention strategies must be person-centred, aligning with the individual’s specific needs and circumstances. Such counselling should remain a core component of the entire care continuum before, during, and after PrEP use.

A multi-country study of PrEP providers [25] explored perceptions of existing and emerging PrEP modalities. Many providers noted the difficulty of administering LAI-PrEP in non-clinical settings due to the technical requirements of injections and the management of potential drug interactions or complex health issues. The study suggests that the clinical demands of LAI-PrEP may lead to the increased medicalization of prophylaxis, potentially restricting access and overburdening health services, particularly smaller clinics with limited supplies and personnel [26]. Within this context, nurses are identified as essential providers for LAI-PrEP delivery. However, there remains significant professional skepticism regarding the assessment and prescription of PrEP by those without specialized training in healthcare. Consequently, peer workers are often restricted to supportive roles rather than clinical procedures, a finding that has major implications for future task-shifting policies and service organization.

## CONCLUSION

Participants expressed mixed views regarding the potential for peer educators to prescribe PrEP. However, while some interviewees believed peer could manage prescriptions, provided they receive adequate training and support, others maintained a strong preference for care delivered by physicians or nurses, valuing the established trust and clinical bond with health professionals. Despite these varied opinions, most participants recognized that peer educators are vital for recognized that peer educators are vital for service engagement and retention. However, the high level of acceptance for nurse and physician-led care has not yet disrupted the prevailing paradigm of PrEP medicalization, even in community settings. This persistence, drug-based, clinical focus undermines the acceptance of the prescription of peer-led prophylaxis. It hinders the consolidation of a broader perspective: that PrEP is a safe intervention, with well-established protocols, where management is largely a matter of acquiring appropriate training. This medicinal perception was even more pronounced regarding LAI-PrEP, despite the irony that long-acting formulations actually reduce the daily burden of drug-taking and clinical monitoring for the user.

A primary limitation of this study is its foundation in a survey of oral PrEP users within a demonstration project, where participants’ only experience was with professional-led prescription. This prior exposure likely shaped their benchmarks for comparing professional care with lay-led follow-up. Furthermore, the overwhelmingly positive evaluations from healthcare professionals regarding peer-led monitoring may be influenced by social desirability bias. Within the context of a demonstration study, professionals may have felt inhibited or feared reprisal, leading them to downplay insecurities or dissatisfaction regarding task-shifting.

In conclusion, this study sought to clarify the barriers to PrEP access for adolescents, analyze perceptions of community-based prescription by nurses and peers, and explore attitudes toward the delivery of LAI-PrEP across different healthcare staff categories. Our findings indicate that trust is the cornerstone of PrEP delivery. While nurses and physicians are widely trusted to provide both oral and injectable modalities, peer educators are currently viewed in a complementary role focused on education and support. The successful rollout of new PrEP modalities will depend on strengthening these collaborative roles and addressing the structural hierarchies inherent in health systems.

## Supplementary Material

Supplementary Files

This is a list of supplementary files associated with this preprint. Click to download.

- Additionalfile1.docx
- Additionalfile2.docx

## Figures and Tables

**Table 1:** Characterization of clients and healthcare staff

Codename	Recategorized color/race	PrEP client or healthcare staff	Gender identity	Sexual orientation	Education	City
Guto	Black	Client	Cisgender man	Homosexual	Incomplete high school	São Paulo, São Paulo, Brazil
Nina	Black	Client	Cisgender man	Homosexual	Incomplete high school	São Paulo, São Paulo, Brazil
Guiga	White	Client	Gender fluid	Homosexual	Incomplete high school	São Paulo, São Paulo, Brazil
Ramom	Black	Client	Cisgender man	Bisexual	Incomplete higher education	São Paulo, São Paulo, Brazil
Geovane	Black	Client	Cisgender man	Homosexual	Incomplete higher education	São Paulo, São Paulo, Brazil
Eder	White	Client	Cisgender man	Homosexual	Complete high school	São Paulo, São Paulo, Brazil
Prince	Black	Client	Cisgender man	Homosexual	Complete high school	São Paulo, São Paulo, Brazil
George	Black	Client	Cisgender man	Homosexual	Incomplete high school	São Paulo, São Paulo, Brazil
Will	White	Client	Cisgender man	Homosexual	Incomplete high school	São Paulo, São Paulo, Brazil
José	White	Client	Cisgender man	Homosexual	Incomplete higher education	São Paulo, São Paulo, Brazil
Lucas	Black	Client	Cisgender man	Transgender	Complete high school	Salvador, Bahia, Brazil
Amon	Black	Client	Cisgender man	Homosexual	Incomplete high school	Salvador, Bahia, Brazil
Vanessa	Black	Client	TrTW	Heterosexual	Incomplete high school	Salvador, Bahia, Brazil
Henrique	Black	Client	Cisgender man	Bisexual	Incomplete higher education	Salvador, Bahia, Brazil
Luana	Black	Client	TrTW	Heterosexual	High school – studying a technical course in management	Salvador, Bahia, Brazil
Irene	Black	Client	TrTW	Heterosexual	Complete high school	Salvador, Bahia, Brazil
Liandro	Black	Client	Cisgender man	“Questioning”	Incomplete higher education	Salvador, Bahia, Brazil
Gustavo	Black	Client	Cisgender man	Homosexual	Incomplete high school	Salvador, Bahia, Brazil
Cleusa	Black	Client	TrTW	Heterosexual	Incomplete high school	Salvador, Bahia, Brazil
Amanda	Black	Client	TrTW	Heterosexual	Incomplete high school	Salvador, Bahia, Brazil
Ricco	Black	Staff	Cisgender man	Bisexual	Higher education (nursing)	São Paulo, São Paulo, Brazil
Gabriela	White	Staff	Cisgender woman	Heterosexual	Higher education (nursing)	São Paulo, São Paulo, Brazil
Eleonora	White	Staff	Cisgender woman	Heterosexual	Higher education (nursing)	São Paulo, São Paulo, Brazil
Diego	Black	Staff	Cisgender man	SI/NR	Higher education (physician)	São Paulo, São Paulo, Brazil
João	Black	Staff	Cisgender man	Homosexual	SI/NR	São Paulo, São Paulo, Brazil
Daniel	Black	Staff	Transgender man	SI/NR	Incomplete higher education	Salvador, Bahia, Brazil
Nice	White	Staff	Cisgender woman	Heterosexual	Higher education (nursing)	Salvador, Bahia, Brazil
Vitória	White	Staff	Cisgender woman	Homossexual	Higher education (nursing)	Salvador, Bahia, Brazil
Samara	White	Staff	Cisgender woman	Bisexual	Higher education (physician)	Salvador, Bahia, Brazil

## Data Availability

Due to the qualitative nature of the study and ethical restrictions related to participant confidentiality, interview transcripts are not publicly available. Anonymized excerpts may be shared upon reasonable request to the De-identified excerpts of the data may be available from the corresponding author upon reasonable request and subject to approval by the relevant ethics committee.

## STRENGTHS AND LIMITATIONS OF THIS STUDY

- This study employed purposive sampling to ensure diversity in gender identity, age, and geographic context across two economically contrasting Brazilian cities.
- The inclusion of both PrEP clients and healthcare providers (nurses, physicians, and peer educators) enabled triangulation of perspectives on PrEP delivery models.
- Application of COREQ guidelines to ensure rigour in qualitative reporting.
- Findings are based on a demonstration project context, which may limit transferability.
- Participants had no direct experience with injectable PrEP, relying on hypothetical scenarios.
